# Association between AISI and mortality among individuals with abdominal obesity: A cohort study

**DOI:** 10.1097/MD.0000000000047478

**Published:** 2026-01-30

**Authors:** Qingmei Niu, Junkang Zhao, Yatian Jia, Shuo Ding, Yuxiang Gao, Wenjing Li, Qian Zhang

**Affiliations:** a Department of Nursing, Shanxi Bethune Hospital, Shanxi Academy of Medical Sciences, Third Hospital of Shanxi Medical University, Tongji Shanxi Hospital, Taiyuan, Shanxi, China; b Department of Scientific Research, Third Hospital of Shanxi Medical University, Shanxi Bethune Hospital, Shanxi Academy of Medical Sciences, Tongji Shanxi Hospital, Taiyuan, China; c School of Nursing, Shanxi University of Chinese Medicine, Jinzhong, China.

**Keywords:** abdominal obesity, AISI, inflammatory effect, mortality

## Abstract

This study examines the association between the aggregate index of systemic inflammation (AISI) and mortality among individuals with abdominal obesity, aiming to provide a scientific basis for accurate identification of high-risk individuals and the implementation of early interventions. This study utilized data from the National Health and Nutrition Examination Survey (NHANES) 1999–2016 to include adults with abdominal obesity. The AISI was calculated as (neutrophil count × platelet count × monocyte count)/lymphocyte count. Participants were linked to the National Death Index to determine vital status, with mortality follow-up extending through December 31, 2019. The association between the AISI and mortality risk in individuals with abdominal obesity was examined through Kaplan–Meier survival analysis, weighted Cox proportional hazards regression, subgroup stratification, and restricted cubic spline modeling. This cohort study enrolled 15,839 adult participants with abdominal obesity. During a median follow-up of 119 months, 2223 deaths were recorded. Kaplan–Meier survival analysis showed that elevated levels of the AISI were significantly associated with reduced survival probability. After comprehensive adjustment for multiple potential confounders, multivariate Cox proportional hazards regression revealed a significant positive association between AISI levels and all-cause mortality risk in individuals with abdominal obesity. When AISI was treated as a continuous variable, each 1-unit increase was associated with a 1% higher risk of death (fully adjusted HR = 1.01, 95% CI: 1.01–1.01, *P* < .001). When dichotomized at the median, the high AISI group exhibited a 16% increased risk of mortality compared to the low AISI group (fully adjusted HR = 1.16, 95% CI: 1.04–1.30, *P* = .010). Restricted cubic spline analysis confirmed a statistically significant linear relationship (*P* for overall < .001; *P* for nonlinearity = .192). Subgroup analyses indicated heterogeneity in this association across marital status subgroups (interaction *P* = .017). This study demonstrates that elevated AISI levels are associated with increased mortality risk among individuals with abdominal obesity. These findings underscore the value of AISI as a prognostic indicator for mortality in this at-risk population. The integration of AISI into clinical inflammatory assessment frameworks may facilitate the establishment of dynamic surveillance strategies for identifying and monitoring high-risk individuals.

## 1. Background

Abdominal obesity, characterized by the pathological accumulation of visceral adipose tissue, constitutes a more detrimental metabolic condition than generalized obesity as defined by body mass index (BMI).^[1]^ Evidence from the World Health Organization (WHO) indicates that individuals with abdominal obesity exhibit significantly higher all-cause mortality rates compared to those with normal waist circumference, yet the underlying pathophysiological mechanisms remain incompletely elucidated.^[2]^ Recent epidemiological data show a prevalence of 31.7% for abdominal obesity based on waist circumference criteria,^[3]^ underscoring its emergence as a major public health challenge. Abdominal obesity has been robustly associated not only with key metabolic disorders – such as type 2 diabetes, hypertension and dyslipidemia – but also with increased susceptibility to cardiovascular disease and certain cancers, thereby contributing directly or indirectly to elevated overall mortality risk.^[4-6]^ Given the escalating global prevalence of abdominal obesity, the development of precise risk prediction tools, timely identification of high-risk individuals, and the implementation of individualized prevention and management strategies are critical for mitigating disease progression and improving long-term health outcomes.

Emerging evidence suggests that visceral adipose tissue actively secretes a wide array of inflammatory mediators, several of which have been implicated in the pathogenesis of obesity-related complications.^[7]^ The progressive accumulation of abdominal fat is associated with increased infiltration of macrophages into adipose depots, leading to elevated production and release of pro-inflammatory cytokines, thereby contributing to the establishment of a chronic, low-grade systemic inflammatory state.^[3]^ Although established biomarkers such as C-reactive protein are consistently correlated with abdominal adiposity,^[8]^ they exhibit significant limitations in clinical utility, as they reflect only isolated components of the inflammatory response and fail to capture the complexity of the underlying inflammatory network in abdominal obesity. Therefore, there is an urgent need for integrative and multifaceted biomarkers capable of enabling a more accurate, comprehensive, and clinically meaningful evaluation of the inflammatory burden associated with this metabolic condition.

The recently introduced aggregate index of systemic inflammation (AISI) dynamically integrates critical immunological components – neutrophil phagocytic function, monocyte migratory capacity, platelet-driven pro-inflammatory activity, and lymphocyte-mediated immune surveillance – demonstrating promising utility in the prediction of cancer progression and clinical outcomes.^[9,10]^ Notably, the hypoxic microenvironment characteristic of abdominal obesity promotes granulocyte colony-stimulating factor secretion, leading to an imbalance in the neutrophil-to-lymphocyte ratio.^[11]^ Furthermore, interleukin-1β derived from visceral adipose tissue activates the Janus kinase 2/signal transducer and activator of transcription 3 signaling cascade, thereby enhancing platelet hyperactivity.^[12]^ These pathophysiological changes correspond directly to the core variables comprising the AISI algorithm, suggesting that AISI may serve as a mechanistic link between adipose inflammation and elevated mortality risk. As such, AISI holds potential as a more accurate and biologically relevant tool for risk stratification in populations with abdominal obesity.

The association between AISI levels and mortality in individuals with abdominal obesity remains insufficiently characterized. To address this knowledge gap, the current study aims to investigate the relationship between AISI and all-cause mortality within this population using data from the National Health and Nutrition Examination Survey (NHANES). Elucidating this association is critical for advancing the clinical applicability of AISI in risk assessment and health management strategies for individuals with abdominal obesity.

## 2. Materials and methods

### 2.1. Study population

**Figure 1.**
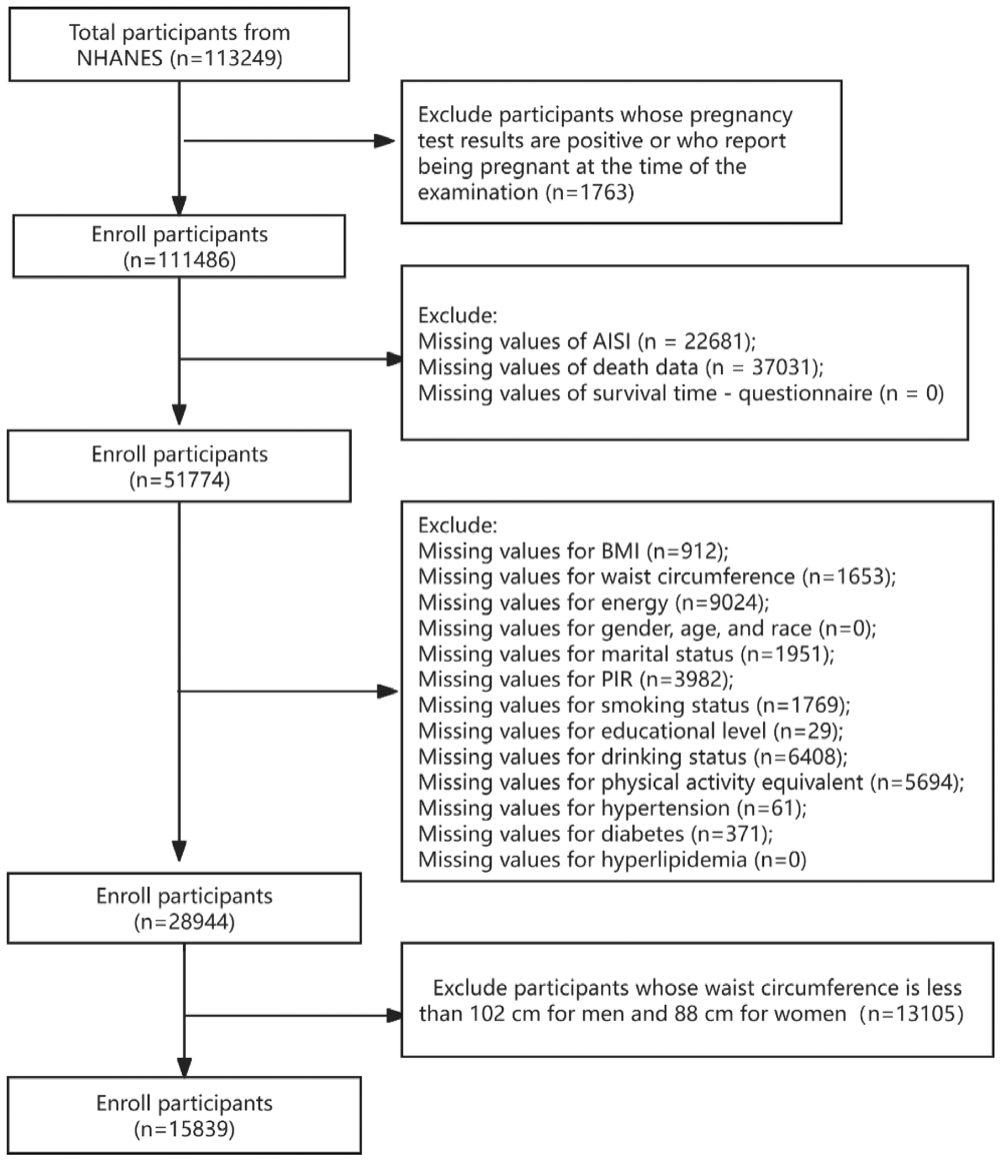
Flowchart of the screening process for the study population. AISI = aggregate index of systemic inflammation, BMI = body mass index, NHANES = the National Health and Nutrition Examination Survey, PIR = family income-to-poverty ratio.

The data used in this study were derived from the NHANES, a nationally representative program administered by the National Center for Health Statistics (NCHS) of the United States. NHANES is an ongoing cross-sectional survey designed to monitor the health and nutritional status of the civilian, noninstitutionalized US population through standardized interviews, physical examinations, and laboratory assessments. Data collected include demographic profiles, dietary intake patterns, clinical examination findings, biochemical laboratory results, and responses to health-related questionnaires. The NHANES protocol was reviewed and approved by the NCHS Institutional Review Board, with all participants providing written informed consent prior to enrollment. Mortality outcomes were ascertained through probabilistic linkage with the National Death Index, a comprehensive database maintained by the Centers for Disease Control and Prevention (https://www.cdc.gov/nchs/data-linkage/mortality-public.htm). The participant selection process is summarized in Figure 1. The analysis included individuals from NHANES cycles conducted between 1999 and 2016 who had complete information on the AISI and abdominal obesity status. Exclusion criteria consisted of pregnancy, insufficient data on mortality, AISI calculation, or essential covariates, as well as failure to meet the established threshold for abdominal obesity – defined as a waist circumference of <102 cm in males or <88 cm in females.

### 2.2. AISI calculation

This study evaluated the AISI, a systemic inflammatory biomarker, using laboratory data derived exclusively from standard complete blood count assays. The AISI was calculated according to the following formula: AISI = (Neutrophil count [NC] × Platelet count [PC] × Monocyte count [MC])/Lymphocyte count [LC].^[13]^

### 2.3. Diagnostic criteria for abdominal obesity

All baseline anthropometric measurements were conducted by trained healthcare professionals following a standardized protocol. Abdominal obesity was defined according to the WHO’s established waist circumference criteria, with thresholds set at ≥88 cm for women and ≥102 cm for men.^[14]^ Waist circumference was measured using a Seca-201 nonelastic tape measure placed horizontally at the midpoint between the inferior margin of the last palpable rib and the superior border of the iliac crest, aligned with the midaxillary line.

### 2.4. Covariates

Based on established literature and clinical criteria,^[15-17]^ the following potential confounders were included in the analysis: age (dichotomized as <60 years and ≥60 years), sex (male or female), race/ethnicity (Mexican American, other Hispanic, non-Hispanic White, non-Hispanic Black, and other races), marital status (married, cohabiting, divorced, widowed, separated, or single), educational attainment (<high school diploma, high school graduate or equivalent, college graduate, or higher), family income-to-poverty ratio (PIR) categorized as <1.30, 1.30–3.49, and≥3.50), BMI (classified as < 25, 25–29.9, and ≥30 kg/m^2^), smoking history (never, former, or current smoker), alcohol consumption (yes/no), and physical activity level, which was stratified into high and low categories. Hypertension was defined as a self-reported diagnosis of hypertension, current use of antihypertensive medication, or measured systolic blood pressure > 140 mm Hg or diastolic blood pressure > 90 mm Hg; meeting any one of these criteria was sufficient for classification. Diabetes was defined by any of the following: previous diagnosis of diabetes, current use of insulin or oral hypoglycemic agents, hemoglobin A1c ≥ 6.5%, fasting plasma glucose ≥ 126 mg/dL, or 2-hour postprandial glucose ≥ 200 mg/dL. Hyperlipidemia was defined as the presence of at least one of the following: triglycerides ≥ 150 mg/dL, total cholesterol ≥ 200 mg/dL, low-density lipoprotein cholesterol ≥ 130 mg/dL, high-density lipoprotein cholesterol ≤ 40 mg/dL in men or ≤ 50 mg/dL in women, or current use of lipid-lowering medications.

### 2.5. Statistical analysis

All statistical analyses were conducted in accordance with the methodological guidelines established by the NHANES of the United States. To ensure representativeness of the US national population and generalizability to the noninstitutionalized civilian population, appropriate sample weights were applied throughout the analytical process. Data processing and statistical modeling were performed using R version 4.4.0 (2024-04-24). Given the complex multistage probability sampling design of NHANES, survey design features – including primary sampling units, stratification, and sampling weights – were incorporated into all analyses using the “survey” package in R. This approach enabled valid population-level inference while accounting for clustering and stratification effects, thereby preventing inflation of type I error rates. Consistent with NHANES recommendations, weight selection prioritized the representation of smaller subgroups to enhance the precision and reliability of subgroup-specific estimates. Weighted estimation techniques were implemented using the “survey” package; survival curves were generated via the “jskm” package, an RCS plots were constructed using the “rms” package. Additional descriptive statistics and graphical outputs were produced using the online platform zstats1.0 (www.zstats.net). Statistical significance was defined as a 2-sided *P* value < .05. For comparisons of baseline characteristics between groups, design-adjusted chi-square tests (for categorical variables) and *t*-tests (for continuous variables) were employed to account for the complex survey structure. To maintain consistency in laboratory measurements across different NHANES cycles, key parameters were harmonized using NHANES-recommended correction factors. Only data meeting stringent internal quality control and external quality assessment standards were included in the final analytic sample. For physical activity assessment, responses from both pre- and post-2007 versions of the questionnaire were standardized into a unified metric – “standardized total physical activity” – expressed in metabolic equivalent minutes per week (MET-min/wk), based on intensity classification criteria set forth by the WHO. The association between AISI and all-cause mortality was assessed using multivariable-adjusted Cox proportional hazards models. Three progressively adjusted models were specified: model 1 was unadjusted; model 2 was adjusted for age, sex, and race/ethnicity; and model 3 further adjusted for marital status, smoking and alcohol consumption, educational attainment, physical activity, BMI, PIR, and prevalent comorbidities including hypertension, diabetes, and hyperlipidemia. Differences in survival probabilities across AISI categories were evaluated using the Kaplan–Meier method, with statistical significance assessed via the log-rank test. In light of prior NHANES-based evidence suggesting a nonlinear relationship between AISI and health outcomes,^[15-17]^ and considering the current study’s sample size and variable distribution, a RCS model with 4 knots was fitted to explore potential nonlinearity in the dose-response relationship between AISI and mortality risk. This modeling strategy allows for flexible curve fitting while minimizing overfitting. In the absence of an established clinical cutoff for AISI, the median value (273) was selected as the reference point, providing a robust and centrally located baseline that is less influenced by outliers. Prespecified subgroup analyses were conducted across key demographic, socioeconomic, and clinical dimensions: sex (male/female), age group (<60 vs ≥60 years), race/ethnicity (Mexican American, non-Hispanic White, non-Hispanic Black, other Hispanic, or multiracial), BMI category (<25, 25–<30, ≥30 kg/m^2^), education level (less than high school, high school graduate, college, or higher), PIR category (<1.30, 1.30–<3.50, ≥3.50), smoking and drinking status (yes/no), physical activity level (high/low), and presence of comorbidities (diabetes, hypertension, or hyperlipidemia). To assess the robustness of the findings, 3 sensitivity analyses were conducted: exclusion of participants who experienced events within the first 2 years of follow-up to reduce potential reverse causality; rerunning the fully adjusted model without including diabetes, hypertension, and hyperlipidemia as covariates to evaluate their role as potential mediators or confounders; and application of multiple imputation techniques – predictive mean matching for continuous variables and logistic regression-based imputation for categorical variables – to handle missing data, thereby reducing selection bias and maintaining statistical power.

## 3. Results

### 3.1. Study population

Between 1999 and 2016, the study initially enrolled 1,13,249 participants. Following the exclusion of 97,410 individuals, a final analytical sample of 15,839 adults with abdominal obesity was established, as illustrated in Figure 1 (the study flow diagram). Baseline demographic and clinical features of this cohort are presented in Table 1. Based on the dichotomous classification of the AISI index, the participants were categorized into 2 groups: 8402 (53.05%) fell into AISI category 1, while 7437 (46.95%) belonged to category 2. The 2 groups showed statistically significant differences in gender distribution, race, marital status, BMI, PIR, smoking behavior, educational attainment, physical activity levels, and the presence of hypertension, diabetes, and hyperlipidemia (all *P*-values < .05). In contrast, age and alcohol use status did not differ significantly between the groups (*P* > .05).

**Table 1:** Baseline characteristics of participants.

Variable	Total (n = 15,839)	AISI ≤ 273 (n = 8402)	AISI > 273 (n = 7437)	Statistic	*P*
Sex (%)				χ^2^ = 19.06	**<.001**
Male	6392 (41.43)	3191 (39.73)	3201 (43.14)		
Female	9447 (58.57)	5211 (60.27)	4236 (56.86)		
Race (%)				χ^2^ = 244.00	**<.001**
Mexican American	2643 (7.28)	1441 (7.62)	1202 (6.95)		
Other Hispanic	1382 (4.85)	720 (4.54)	662 (5.17)		
Non-Hispanic White	7815 (73.95)	3594 (70.14)	4221 (77.75)		
Non-Hispanic Black	3232 (10.03)	2236 (13.65)	996 (6.42)		
Other Race	767 (3.88)	411 (4.05)	356 (3.71)		
Marital status (%)				χ^2^ = 32.15	**.001**
Married	8641 (60.24)	4627 (61.89)	4014 (58.59)		
Widowed	1512 (6.62)	760 (5.97)	752 (7.27)		
Divorced	1943 (11.44)	1042 (11.06)	901 (11.82)		
Separated	521 (2.45)	267 (2.02)	254 (2.87)		
Never married	2209 (13.15)	1155 (12.89)	1054 (13.41)		
Living with partner	1013 (6.11)	551 (6.17)	462 (6.04)		
Smoking status (%)				χ^2^ = 103.58	**<.001**
Never	8539 (53.02)	4820 (56.57)	3719 (49.48)		
Former	4434 (28.64)	2248 (27.77)	2186 (29.52)		
Now	2866 (18.33)	1334 (15.66)	1532 (21.01)		
Education (%)				χ^2^ = 22.92	**.001**
Less than high school	3797 (15.13)	2085 (15.40)	1712 (14.87)		
High school or equivalent	3822 (24.49)	1928 (22.85)	1894 (26.12)		
College or above	8220 (60.38)	4389 (61.75)	3831 (59.01)		
Alcohol status (%)				χ^2^ = 3.82	.100
No	4904 (25.96)	2741 (26.64)	2163 (25.28)		
Yes	10,935 (74.04)	5661 (73.36)	5274 (74.72)		
Metabolic equivalent of task (MET; %)				χ^2^ = 31.65	**<.001**
Low physical activity	6789 (39.50)	3504 (37.32)	3285 (41.69)		
High physical activity	9050 (60.50)	4898 (62.68)	4152 (58.31)		
Hypertension (%)				χ^2^ = 31.91	**<.001**
No	7590 (52.99)	4172 (55.23)	3418 (50.75)		
Yes	8249 (47.01)	4230 (44.77)	4019 (49.25)		
Diabetes (%)				χ^2^ = 20.47	**<.001**
No	12,057 (82.24)	6453 (83.62)	5604 (80.87)		
Yes	3782 (17.76)	1949 (16.38)	1833 (19.13)		
Hyperlipidemia (%)				χ^2^ = 22.30	**<.001**
No	3035 (18.81)	1728 (20.27)	1307 (17.34)		
Yes	12,804 (81.19)	6674 (79.73)	6130 (82.66)		
Age (%)				χ^2^ = 0.83	.463
<60 yr old	9757 (71.09)	5167 (71.42)	4590 (70.76)		
≥60 yr old	6082 (28.91)	3235 (28.58)	2847 (29.24)		
BMI (%)				χ^2^ = 57.72	**<.001**
<25	732 (5.02)	436 (5.89)	296 (4.14)		
25 ≤ BMI < 30	5114 (33.05)	2820 (34.84)	2294 (31.25)		
≥30	9993 (61.93)	5146 (59.27)	4847 (64.60)		
PIR (%)				χ^2^ = 12.90	**.014**
<1.3	4726 (19.37)	2500 (19.11)	2226 (19.63)		
1.3 ≤ PIR < 3.5	6049 (35.92)	3158 (34.79)	2891 (37.04)		
≥3.5	5064 (44.72)	2744 (46.10)	2320 (43.33)		

Bold values represent statistically significant results, with the following significance levels: *P* < .05, *P* < .01, and *P* < .001.

χ^2^ = Chi-square test, AISI = aggregate index of systemic inflammation, BMI = body mass index, MET = metabolic equivalent of task, PIR = family income-to-poverty ratio.

### 3.2. Relationship between AISI index and mortality in patients with abdominal obesity

As shown in Table 2, weighted multivariate Cox proportional hazards regression analysis demonstrated a statistically significant positive association between AISI levels and mortality risk among individuals with abdominal obesity, with the association remaining robust across progressively adjusted models. In the crude model (model 1), participants in the second quartile (Q2) of AISI had a 30% higher risk of death compared to those in the lowest quartile (Q1;HR = 1.30, 95% CI: 1.15–1.47, *P* < .001). Following adjustment for demographic variables – age, sex, and race – in model 2, the hazard ratio was marginally attenuated (HR = 1.29, 95% CI: 1.14–1.46, *P* < .001). Further adjustment in model 3 for BMI, lifestyle factors (smoking, alcohol consumption, physical activity), socioeconomic status (education, income-to-poverty ratio), and metabolic comorbidities (hypertension, diabetes, hyperlipidemia) resulted in additional attenuation of the effect estimate (HR = 1.16, 95% CI: 1.04–1.30, *P* = .010), although the association remained statistically significant. The approximately 45% reduction in the hazard ratio from model 2 to model 3 suggests that these intermediate factors may partially mediate the relationship between AISI and mortality. Nonetheless, AISI remains an independent predictor of all-cause mortality in this population.

**Table 2:** Weighted association between AISI and mortality among individuals with abdominal obesity: findings from multivariable Cox proportional hazards regression analysis.

Variables	Model 1		Model 2		Model 3	
	HR (95% CI)	*P*	HR (95%CI)	*P*	HR (95%CI)	*P*
AISI	1.01 (1.01–1.01)	**<.001**	1.01 (1.01–1.01)	**<.001**	1.01 (1.01–1.01)	**<.001**
AISI median						
1	1.00 (Reference)		1.00 (Reference)		1.00 (Reference)	
2	1.30 (1.15–1.47)	**<.001**	1.29 (1.14–1.46)	**<.001**	1.16 (1.04–1.30)	**.010**

Model 1: Crude.

Model 2: Adjust: Sex, race, age.

Model 3: Adjust: Sex, race, age, BMI, marital status, education, PIR, energy intake, smoking status, alcohol consumption, physical activity equivalent, hypertension, diabetes, hyperlipidemia.

Bold values represent statistically significant results, with the following significance levels: *P* < .05, *P* < .01, and *P* < .001.

AISI = aggregate index of systemic inflammation, BMI = body mass index, CI = confidence Interval, HR = hazard ratio, PIR = family income-to-poverty ratio.

**Figure 2.**
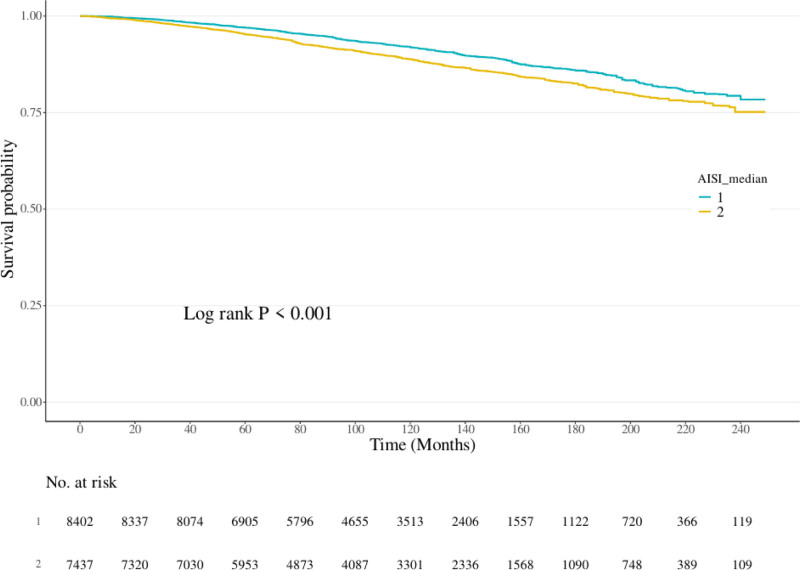
Kaplan–Meier survival curves for all-cause mortality in individuals with abdominal obesity stratified by AISI quartiles. AISI = aggregate index of systemic inflammation.

Over the course of the study, a total of 2223 deaths were documented. Kaplan–Meier survival analysis revealed statistically significant differences in survival probabilities among individuals with abdominal obesity when stratified by median AISI levels (log-rank test, *P* < .001), as depicted in Figure 2. Higher AISI values were consistently associated with poorer survival outcomes. Notably, participants in the upper quartiles of AISI exhibited a greater risk of mortality compared to those in the lower quartiles.

**Figure 3.**
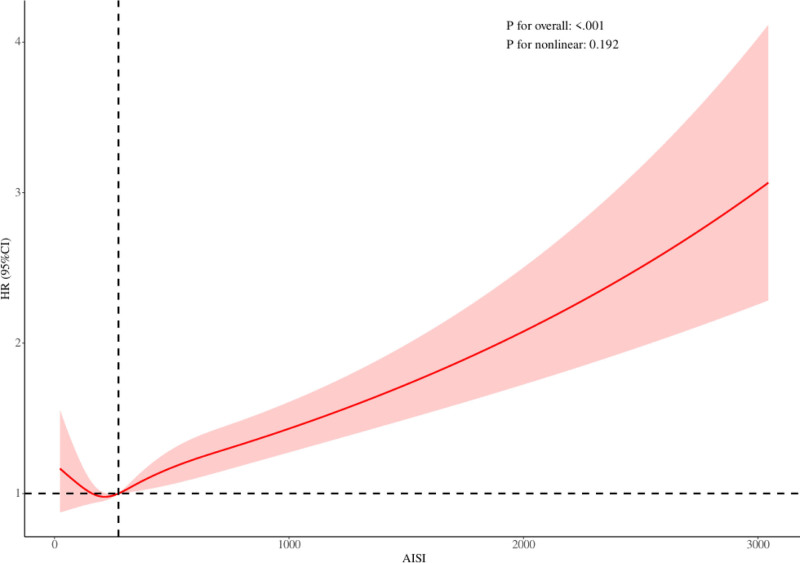
The association between AISI and mortality among individuals with abdominal obesity was examined using a restricted cubic spline model. AISI = aggregate index of systemic inflammation, CI = confidence interval, HR = hazard ratio.

The RCS analysis presented in Figure 3 indicates a statistically significant association between AISI scores and mortality risk among individuals with abdominal obesity (*P* for overall < .001), with the relationship exhibiting a predominantly linear pattern (*P* for nonlinearity = .192). A progressive increase in the hazard ratio for mortality was observed as AISI values increased within this population.

A subgroup analysis of 15,839 individuals with abdominal obesity demonstrated that an elevated systemic immune-inflammation index (AISI > 273) is significantly associated with increased mortality risk in the overall cohort (HR = 1.16, *P* = .010; see Table 3). Marital status was identified as a significant effect modifier (interaction *P* = .017), with widowed individuals exhibiting a substantially higher risk (HR = 1.59, *P* < .001), while no statistically significant increase in risk was observed among divorced individuals. This finding suggests a meaningful interaction between social factors and inflammatory pathways in influencing health outcomes. In contrast, no significant effect modification was detected across subgroups defined by sex, race, smoking status, alcohol consumption, educational attainment, physical activity level, comorbid conditions (including hypertension, diabetes, and hyperlipidemia), age, socioeconomic status, or BMI (all interaction *P* > .05), indicating that the association between AISI and mortality risk is consistent and robust across diverse demographic and clinical populations.

**Table 3:** Subgroup analysis of the association between the AISI and mortality among Individuals with abdominal obesity.

Subgroup	n (%)	AISI ≤ 273	AISI > 273	HR (95% CI)	*P*	*P* for interaction
All patients	15,839 (100.00)	1000/8402	1223/7437	1.16 (1.04–1.30)	**.010**	
Sex						.246
Male	6392 (40.36)	482/3191	687/3201	1.22 (1.04–1.43)	**.014**	
Female	9447 (59.64)	518/5211	536/4236	1.11 (0.96–1.29)	.157	
Race						.456
Mexican American	2643 (16.69)	120/1441	106/1202	1.08 (0.71–1.64)	.715	
Other Hispanic	1382 (8.73)	59/720	62/662	1.22 (0.74–2.00)	.443	
Non-Hispanic White	7815 (49.34)	571/3594	887/4221	1.12 (0.98–1.27)	.085	
Non-Hispanic Black	3232 (20.41)	225/2236	135/996	1.50 (1.18–1.89)	**<.001**	
Other Race	767 (4.84)	25/411	33/356	1.72 (0.88–3.36)	.113	
Marital status						.017
Married	8641 (54.56)	526/4627	625/4014	1.18 (1.01–1.37)	**.034**	
Widowed	1512 (9.55)	222/760	327/752	1.59 (1.27–1.98)	**<.001**	
Divorced	1943 (12.27)	143/1042	136/901	0.72 (0.51–1.01)	.055	
Separated	521 (3.29)	17/267	26/254	1.99 (0.92–4.28)	.079	
Never married	2209 (13.95)	70/1155	71/1054	0.93 (0.61–1.40)	.718	
Living with partner	1013 (6.40)	22/551	38/462	1.90 (0.96–3.75)	.064	
Smoking status						.487
Never	8539 (53.91)	432/4820	445/3719	1.19 (0.96–1.47)	.104	
Former	4434 (27.99)	411/2248	557/2186	1.22 (1.05–1.43)	**.010**	
Now	2866 (18.09)	157/1334	221/1532	1.04 (0.80–1.37)	.759	
Education						.232
Less than high school	3797 (23.97)	367/2085	374/1712	1.07 (0.86–1.34)	.537	
High school or equivalent	3822 (24.13)	232/1928	329/1894	1.41 (1.11–1.80)	**.006**	
College or above	8220 (51.90)	401/4389	520/3831	1.05 (0.89–1.24)	.582	
Alcohol status						.904
No	4904 (30.96)	368/2741	382/2163	1.16 (0.94–1.43)	.163	
Yes	10,935 (69.04)	632/5661	841/5274	1.16 (1.00–1.34)	.054	
Metabolic equivalent of task (MET)						.230
Low physical activity	6789 (42.86)	527/3504	665/3285	1.24 (1.06–1.45)	**.007**	
High physical activity	9050 (57.14)	473/4898	558/4152	1.10 (0.92–1.31)	.305	
Hypertension						.926
No	7590 (47.92)	249/4172	264/3418	1.14 (0.92–1.41)	.218	
Yes	8249 (52.08)	751/4230	959/4019	1.17 (1.02–1.35)	**.028**	
Diabetes						.466
No	12,057 (76.12)	597/6453	740/5604	1.21 (1.04–1.40)	**.015**	
Yes	3782 (23.88)	403/1949	483/1833	1.08 (0.88–1.31)	.457	
Hyperlipidemia						.289
No	3035 (19.16)	148/1728	171/1307	1.34 (0.97–1.86)	.074	
Yes	12,804 (80.84)	852/6674	1052/6130	1.13 (1.00–1.28)	.057	
Age						.733
<60 yr old	9757 (61.60)	196/5167	215/4590	1.10 (0.85–1.41)	.471	
≥60 yr old	6082 (38.40)	804/3235	1008/2847	1.19 (1.05–1.34)	**.007**	
PIR						.545
<1.3	4726 (29.84)	343/2500	384/2226	1.16 (0.92–1.46)	.222	
1.3 ≤ PIR < 3.5	6049 (38.19)	437/3158	537/2891	1.08 (0.91–1.28)	.370	
≥3.5	5064 (31.97)	220/2744	302/2320	1.29 (1.01–1.66)	**.041**	
BMI						.756
<25	732 (4.62)	76/436	80/296	1.38 (0.91–2.10)	.132	
25 ≤ BMI < 30	5114 (32.29)	394/2820	483/2294	1.17 (0.94–1.45)	.154	
≥30	9993 (63.09)	530/5146	660/4847	1.15 (0.99–1.35)	.071	

Bold values represent statistically significant results, with the following significance levels: *P* < .05, *P* < .01, and *P* < .001.

CI = confidence interval, BMI = body mass index, HR = hazard ratio, MET = metabolic equivalent of task, PIR = family income-to-poverty ratio.

Sensitivity analyses confirmed that the results remained stable after excluding subjects from the initial year of follow-up and those with metabolic disorders, including hypertension, diabetes, and hyperlipidemia. Furthermore, imputation of missing covariate data – using predictive mean matching for continuous variables and logistic regression for categorical variables – did not affect the observed trends. Detailed information is available in Tables 4–6.

**Table 4:** Results of the Cox multivariate regression analysis with covariate adjustment.

Variables	Model 1		Model 2	
	HR (95% CI)	*P*	HR (95% CI)	*P*
AISI median				
1	1.00 (Reference)		1.00 (Reference)	
2	1.32 (1.20–1.44)	**<.001**	1.24 (1.12–1.37)	**<.001**

Model 1: Crude.

Model 2: Adjust: Sex, race, age, BMI, marital status, education, PIR, energy intake, smoking status, alcohol consumption, physical activity equivalent, hypertension, diabetes, hyperlipidemia.

Bold values represent statistically significant results, with the following significance levels: *P* < .05, *P* < .01, and *P* < .001.

AISI = aggregate index of systemic inflammation, BMI = body mass index, CI: = confidencei, HR = hazard ratio, PIR = family income-to-poverty ratio.

**Table 5:** Findings from the Cox multivariate regression analysis after the exclusion of metabolic disorders.

Variables	Model 1		Model 2	
	HR (95% CI)	*P*	HR (95% CI)	*P*
AISI median				
1	1.00 (Reference)		1.00 (Reference)	
2	1.30 (1.15–1.47)	**<.001**	1.20 (1.06–1.35)	**.004**

Model 1: Crude.

Model 2: Adjust: Sex, race, age, BMI, marital status, education, PIR, energy intake, smoking status, alcohol consumption, physical activity equivalent.

Bold values represent statistically significant results, with the following significance levels: *P* < .05, *P* < .01, and *P* < .001.

AISI = aggregate index of systemic inflammation, BMI = body mass index, CI = confidence interval, HR = hazard ratio, PIR = family income-to-poverty ratio.

**Table 6:** Findings from the Cox multivariate regression analysis performed subsequent to excluding individuals with <2 years of follow-up.

Variables	Model 1		Model 2	
	HR (95% CI)	*P*	HR (95% CI)	*P*
AISI median				
1	1.00 (Reference)		1.00 (Reference)	
2	1.40 (1.29–1.52)	**<.001**	1.20 (1.10–1.30)	**<.001**

Model 1: Crude.

Model 2: Adjust: Sex, race, age, BMI, marital status, education, PIR, energy intake, smoking status, alcohol consumption, physical activity equivalent, hypertension, diabetes, hyperlipidemia.

Bold values represent statistically significant results, with the following significance levels: *P* < .05, *P* < .01, and *P* < .001.

AISI = aggregate index of systemic inflammation, BMI = body mass index, CI = confidence interval, HR = hazard ratio, PIR = family income-to-poverty ratio.

## 4. Discussion

This study, utilizing data from the NHANES, represents the first evidence of a linear association between AISI and mortality risk in individuals with abdominal obesity. The Cox proportional hazards model demonstrated a statistically significant positive relationship, with each 1-unit increase in AISI corresponding to a 1% higher risk of all-cause mortality. These findings were corroborated by Kaplan–Meier survival analysis, which revealed improved survival probabilities among individuals with lower AISI levels. Furthermore, RCS modeling confirmed a significant overall association between AISI and mortality, with a predominantly linear trend, indicating a continuous elevation in mortality risk as AISI values rise. Collectively, these results highlight the potential utility of AISI as a prognostic indicator for all-cause mortality in this population, providing a quantitative foundation for clinical risk assessment and supporting the development of more targeted and individualized management strategies for high-risk patients with abdominal obesity.

A growing body of evidence has employed NHANES data to explore the association between AISI and the risk of various chronic diseases. Huang et al reported a significant positive correlation between AISI and the prevalence of chronic kidney disease,^[17]^ with individuals in the highest tertile of AISI demonstrating a 36% increased odds of disease compared to those in the lowest tertile. Duan et al similarly found that higher AISI levels were significantly associated with an elevated risk of depression when comparing the top and bottom quartiles.^[16]^ Su et al observed that increased AISI was linked to a greater likelihood of type 2 diabetes,^[18]^ while Zhang et al identified a statistically significant positive association between AISI and the incidence of fatty liver disease.^[19]^ In addition, multiple studies have examined the prognostic value of AISI in relation to mortality across distinct clinical populations. Xiu et al demonstrated that elevated AISI levels were independently associated with higher cardiovascular mortality risk among individuals with hypertension.^[20]^ In patients with acute myocardial infarction, Jiang et al reported that increased AISI was significantly predictive of adverse cardiovascular outcomes.^[21]^ Yang et al found that female cancer patients with higher AISI values had significantly increased risks of both all-cause and cardiovascular-specific mortality.^[22]^ Yan et al further showed that peritoneal dialysis patients exhibiting elevated AISI levels faced a greater hazard of cardiovascular death.^[23]^ This study further demonstrated that individuals in the higher AISI category are associated with a significantly elevated risk of mortality, consistent with evidence from previous investigations. Collectively, these results highlight the critical role of chronic inflammation control in preserving inflammatory homeostasis and improving clinical outcomes in individuals with abdominal obesity.

The development and progression of abdominal obesity are multifactorial, with chronic low-grade inflammation recognized as a central pathophysiological mechanism.^[24]^ Visceral adipose tissue, in particular, functions as an active endocrine organ that secretes pro-inflammatory cytokines such as tumor necrosis factor-α and interleukin-6.^[25]^ These mediators stimulate the recruitment and activation of immune cells – including neutrophils and monocytes/macrophages – within adipose depots,^[26]^ thereby amplifying local and systemic inflammatory responses and contributing to the development of insulin resistance, as reflected by elevated AISI levels. In addition, these cytokines interfere with the insulin signaling pathway, leading to impaired insulin sensitivity,^[27]^ while concurrently disrupting lipolytic processes and promoting ectopic lipid redistribution. This metabolic dysregulation is frequently accompanied by a dyslipidemic profile marked by hypertriglyceridemia and reduced high-density lipoprotein cholesterol, which further drives the selective expansion of visceral fat mass.^[28]^ Research has demonstrated that immune cell infiltration, particularly by neutrophils and monocytes, into visceral adipose tissue represents an early event in obesity pathogenesis.^[29]^ Components of AISI may thus actively participate in shaping the adipose tissue inflammatory microenvironment through the release of proteases, reactive oxygen species, and other inflammatory effectors, promoting adipocyte hypertrophy and pericellular fibrosis – structural alterations that exacerbate metabolic dysfunction. This biological cascade may serve as a potential mechanistic explanation for the observed positive association between elevated AISI levels and increased mortality risk among individuals with abdominal obesity.

Subgroup analyses demonstrated that, among individuals with abdominal obesity, AISI is significantly associated with an increased risk of mortality, with marital status identified as a key effect modifier. Widowed individuals exhibited the highest risk, which may be attributed to elevated systemic inflammation resulting from prolonged social and psychological stress.^[30]^ In contrast, no significant interaction was observed for demographic or lifestyle factors such as sex, age, or smoking status, indicating that the prognostic value of AISI as a marker of systemic inflammation is consistent and robust across diverse population subgroups.

This study presents several notable strengths. First, it leverages a large and nationally representative sample from the NHANES, with rigorous application of survey-specific sampling weights and design variables – such as primary sampling units and strata – ensuring valid variance estimation and enhancing the external validity of the results for the noninstitutionalized US civilian population. Second, by utilizing AISI as a composite biomarker rather than relying on individual inflammatory markers, the analysis captures a more comprehensive and biologically integrated profile of systemic inflammation and immune dysregulation, thereby offering improved insight into its association with mortality risk in individuals with abdominal obesity. Nonetheless, several limitations warrant consideration. As an observational cohort study, the design inherently limits causal inference; although extensive multivariable adjustments were made for sociodemographic, lifestyle, and clinical covariates, residual or unmeasured confounding may still influence the observed associations. Furthermore, the generalizability of the findings may be constrained by the exclusive use of US-based data, which may not fully reflect the epidemiological, cultural, or healthcare contexts of populations in other countries or with different racial/ethnic compositions. Therefore, future prospective longitudinal studies in diverse populations are necessary to validate the observed associations, confirm the causal role of AISI in mortality risk stratification, and further elucidate the underlying pathophysiological pathways linking systemic inflammation to adverse outcomes in the context of abdominal obesity.

## 5. Conclusion

This study provides substantial epidemiological evidence suggesting that the AISI may function as a reliable predictor of mortality risk in individuals with abdominal obesity. Further investigation is required to assess its clinical applicability and integration into routine practice. These findings have the potential to support healthcare providers in identifying high-risk patients and personalizing anti-inflammatory therapeutic approaches, thus contributing to improved health outcomes in populations affected by abdominal obesity.

## Author contributions

**Conceptualization:** Qingmei Niu, Junkang Zhao, Yatian Jia, Shuo Ding, Yuxiang Gao, Qian Zhang.

**Data curation:** Qingmei Niu, Junkang Zhao, Yatian Jia, Shuo Ding, Yuxiang Gao, Wenjing Li, Qian Zhang.

**Formal analysis:** Qingmei Niu, Junkang Zhao, Yatian Jia, Shuo Ding, Yuxiang Gao, Wenjing Li, Qian Zhang.

**Investigation:** Qingmei Niu, Junkang Zhao, Qian Zhang.

**Methodology:** Qingmei Niu, Junkang Zhao, Qian Zhang.

**Project administration:** Junkang Zhao, Wenjing Li, Qian Zhang.

**Resources:** Junkang Zhao, Wenjing Li, Qian Zhang.

**Software:** Junkang Zhao, Yuxiang Gao, Wenjing Li, Qian Zhang.

**Supervision:** Qingmei Niu, Junkang Zhao, Yatian Jia, Shuo Ding, Yuxiang Gao, Qian Zhang.

**Validation:** Qingmei Niu, Yuxiang Gao.

**Visualization:** Qingmei Niu, Yuxiang Gao.

**Writing – original draft:** Qingmei Niu, Qian Zhang.

**Writing – review & editing:** Qingmei Niu, Junkang Zhao, Yatian Jia, Shuo Ding, Yuxiang Gao, Wenjing Li, Qian Zhang.
